# Developing a scoring tool for assessing adherence to morbidity management and disability prevention (MMDP) practices in patients with filarial lymphedema: A pilot study

**DOI:** 10.1371/journal.pone.0358191

**Published:** 2026-09-30

**Authors:** Ganga Ramar, Anbusivam Sadhasivam, Vijayalakshmi Gnanasekaran, Madhumidha Periyathambi, Vijayakumar Balakrishnan, Vijesh Sreedhar Kuttiatt

**Affiliations:** 1 Unit of Clinical and Molecular Medicine, ICMR-National Institute for Vector Control Research (NIVCR), Indira Nagar, Puducherry, India; 2 Model Rural Health Research Unit (MRHRU), Karikalampakkam CHC Campus, Puducherry, India; Kazi Nazrul University, INDIA

## Abstract

**Background:**

Morbidity Management and Disability Prevention (MMDP) practices are essential in mitigating the progression of lymphedema and in preventing episodes of adenolymphangitis. Despite their importance, patients’ adherence to these prescribed practices has frequently been found to be suboptimal. Currently, there is a lack of a standardized tool for the objective assessment of patients’ adherence to MMDP practices in patients with filarial lymphedema. This study was undertaken to develop a scoring system for assessing patients’ adherence to MMDP and to validate this among patients attending a filariasis management clinic.

**Methodology:**

A scoring tool (VCRC MMDP Adherence Assessment Tool and VAAT score) was developed to evaluate adherence to MMDP practices across eight defined domains. The total score ranges from 0 to 40, reflecting the degree of adherence. This tool was validated through a cross-sectional study involving 90 patients diagnosed with varying grades of filarial lymphedema.

**Results:**

Based on the VAAT score, patients were categorizable into three different levels of adherence: optimal adherence (≥ 30), medium adherence (20–29) and poor adherence (< 20). Among the 90 patients assessed, 21 (23%) demonstrated optimal adherence, 47 (52%) showed medium adherence and 22 (25%) were categorized as having poor adherence.

**Conclusion:**

This study marks the first development and validation of a scoring system to assess adherence to MMDP practices in patients with filarial lymphedema. The tool proved useful in effectively identifying individuals with suboptimal adherence. However, further validation is required in a greater number of patients in a prospective manner and in different geographical and epidemiological settings.

## Introduction

Lymphatic filariasis (LF) is a neglected tropical disease (NTD) which is the second most common cause of long-term disability worldwide [1]. It is a public health problem with a significant social and economic impact in endemic countries [2]. In India, next to malaria, filariasis has been a major vector-borne disease problem [3]. Major clinical manifestations of LF are limb lymphedema and hydrocele. India alone accounts for 40% of the global burden of filariasis [4]. The global baseline estimates of people affected by lymphatic filariasis were 25 million men with hydrocele and over 15 million people with lymphedema [1]. Considering its public health significance, WHO initiated the Global Programme to Eliminate Lymphatic Filariasis (GPELF). Mass Drug Administration (MDA) to interrupt transmission of infection and Morbidity Management and Disability Prevention (MMDP) catering to those affected with lymphedema and hydrocele are the two pillars of this program [5–7]. Patients with lymphedema often suffer from frequent skin and soft tissue infections termed adenolymphangitis (ADL) [6]. Each episode of ADL adds to disease progression. MMDP program for lymphedema is mainly focused on home-based supportive care. Maintenance of good hygiene of the affected limb prevents ADL attacks and slows down the disease progression [8,9]. Components of MMDP include limb washing with soap and water, limb elevation, limb exercises, wearing footwear, application of crepe bandages or compression stockings, massaging, nail care and application of liquid paraffin [9–11]. Studies show that patients who adhere to limb hygiene practices suffer from reduced frequency of infection. However, patients often do not adhere to the advised measures, which results in frequent ADL attacks and rapid disease progression [11]. Currently, there are no standard scoring tools for the objective assessment of adherence to MMDP practices in individual patients. In this study, we developed a scoring system (VCRC MMDP Adherence Assessment Tool and the ‘VAAT’ Score) to assess the adherence of patients to prescribed MMDP practices and validated this among patients attending a filarial lymphedema management clinic.

## Methodology

### Development of the scoring system

To develop the scoring tool, the MMDP components were divided into 8 domains of practices, which include limb hygiene, limb elevation, limb exercise, wearing appropriate footwear, wearing crepe bandages, massaging, nail care and application of liquid paraffin. A questionnaire was developed incorporating the eight components, with questions under each domain designed to assess the level of practice and adherence. A weighted scoring system was adopted for the scale, with the domains recognized to be more important having a higher domain score, based on literature and expert consensus generated through a Delphi round. Greater weightage was given to three domains, viz. limb hygiene, exercise and massaging, which are considered foundational to morbidity management and have a direct impact on patient outcomes. These practices require sustained daily effort and have a direct role in improving lymphatic drainage and skin integrity. The questionnaire was pretested among 10 patients with filarial lymphedema who were trained on MMDP practices. The final tool contained 36 structured questions across 8 domains with a total score of 40 (Table 1 and S1 Table in S1 File). Cronbach’s alpha during the pretest phase was 0.799, suggesting a strong internal consistency. Questions were dichotomous, with a score of 0 for not practicing and 1 for practicing. However, four questions tracking the frequency of practice within a day were scored as zero for not practicing and 1 and 2 for practicing once or more than once a day, respectively, to provide weightage for better practice. The survey was administered as a structured interview, with patients asked to report their adherence according to their usual practices over the past 7 days. The tool was named the **VCRC MMDP Adherence Assessment Tool** and the corresponding score was referred to as the **VAAT score**. Based on the VAAT score obtained out of a maximum of 40, three levels of adherence were defined: **optimal adherence** (≥ 30; ≥ 75%), **medium adherence** (20–29; ≥ 50% to < 75%), and **poor adherence** (< 20; < 50%). The cut-offs for categorizing adherence using the VAAT score were established based on an evaluation by a public health professional with experience in LF morbidity management. Optimal adherence was defined as a score of ≥30 out of 40, corresponding to at least 75% adherence, which is conventionally regarded as satisfactory or high compliance in health behaviour studies. Medium adherence was set at 20–29 out of 40 (≥50% but <75%), reflecting moderate practice of recommended measures, while poor adherence was considered as a score below 20 (<50%), indicating insufficient adherence in most domains. These percentage thresholds were determined to align the score interpretation with levels of compliance that have clinical relevance for effective MMDP practices based on similar studies, allowing distinct classification of patients who are highly adherent, moderately adherent, or at risk due to suboptimal adherence.

**Table 1:** Domain-wise scores for the MMDP practices.

S. No	MMDP practices	Domain code	Score allocation
1.	Limb hygiene	LH	12
2.	Limb elevation	LE	2
3.	Limb exercise	LEX	8
4.	Wearing appropriate footwear	LF	3
5.	Wearing crepe bandage/ stockings	LC	4
6.	Massaging	LM	6
7.	Nail care	LN	2
8.	Application of liquid paraffin	LL	3
	**Total**		**40**

### Validation of the scoring system

#### Study design and location.

A cross-sectional study was undertaken to validate the scoring system at the Filariasis Management Clinic located in ICMR – National Institute for Vector Control Research (NIVCR) (formerly VCRC), Puducherry during 07/09/2022–14/10/2022. The study was approved by the NIVCR Institute Human Ethics Committee, and written informed consent was obtained from the participants.

#### Study participants.

The study population included patients visiting the Filariasis Management Clinic who were trained on MMDP and practicing the same for at least two months. Participants who were not trained and practicing MMDP for 2 months were excluded from the study. The questionnaire was administered by a trained public health professional who was independent of the treating team to minimize response bias due to social desirability or perceived expectations. Consecutive patients attending the clinic were approached, and consenting individuals were enrolled. The study questionnaire was administered to the participants and scores in each domain were recorded and the total score was calculated.

#### Sample size calculation and statistical analysis.

Since the study was designed as an exploratory pilot project, 30 consenting participants with a lymphedema grade of 2, 3 or 4 each were enrolled purposively. Data were entered in a Microsoft Excel spreadsheet, and the statistical analysis was carried out using IBM SPSS 16.0. Frequencies with percentages and mean with standard deviation were used to express summary information of categorical and continuous variables, respectively. Participants were categorized based on the total score and also on the basis of adherence score under each domain of MMDP practices. Adherence and association with socio- demographic and clinical variables were analysed using the chi-square test. P-value less than 0.05 is considered significant.

## Results

The VCRC MMDP Adherence Assessment Tool was validated among a total of 90 patients, which included 30 patients each with lymphedema grade 2, 3, and 4 (S2 File). 48 (53.3%) of patients had lymphedema in the left lower limb, 38 (42.2%) had lymphedema in the right lower limb, and 4 (4.4%) had lymphedema in both lower limbs. There were 25 male and 65 female participants. 48 patients were below or equal to 60 years of age. Majority of the patients were unemployed (n = 65). 48 patients were not suffering from any comorbidities. Clinical and demographic details were consolidated in Table 2.

**Table 2:** Characteristics of the participants.

Characteristics	Frequency (N=90)	Percentage
**Gender**		
Male	25	27.8
Female	65	72.2
**Age**		
>60 years	48	51.1
≤ 60 years	42	48.9
**Education**		
Below Secondary	46	72.2
Secondary and above	44	27.8
**Occupation**		
Unemployed	65	65.6
Employed	25	34.4
**Grades of lymphedema**		
Grade Ⅱ	30	33.3
Grade Ⅲ	30	33.3
Grade IV	30	33.3
**Affected body part**		
Right lower limb	38	42.2
Left lower limb	48	53.3
Bilateral	4	4.4
**No. of visits to clinic in the previous year**		
≤ 3 times	61	67.8
> 3 times	29	32.2
Comorbidity*		
Yes	42	46.7
No	48	53.3

*Comorbidities include diabetes, hypertension, thyroid and cardiac diseases.

The mean VAAT score was found to be 24.46 (±6.09), indicating that the participant cohort had a medium level of adherence. Based on the three categories of adherence of the VAAT score, 21(23%) patients were found to have optimal adherence, 47(52%) showed medium level and 22(25%) showed poor adherence.

Adherence to practices in each of the domains were also categorizable into the same three different levels. More than 80% of the patients showed optimal adherence in the domains of limb hygiene (86.7%), use of appropriate footwear (80%) and nail care (86.7%). But only lesser number of participants had optimal adherence in certain domains like wearing crepe bandages or stockings (58.9%), massaging (56.7%) and application of liquid paraffin (43.3%).

Next, we analyzed the various possible factors associated with different levels of adherence to MMDP practices (Table 3). Age, gender, occupation, education, marital status, grade of lymphedema, and co-morbid conditions were not significantly associated with adherence to MMDP practices. However, a higher VAAT score was significantly associated with the frequency of clinic visits (p = 0.015).

**Table 3:** Factors associated with adherence to MMDP practices.

Sociodemographic and clinical characteristics	Optimal adherence(n%)	Medium level of adherence(n%)	Poor adherence(n%)	P-value
**Gender** Male	9(36.0)	13(52.0)	3(12.0)	0.102
Female	12(18.5)	34(52.3)	19(29.2)	
**Age** >60 years	12(25.0)	25(52.1)	11(22.9)	0.895
≤ 60 years	9(21.4)	22(52.4)	11(26.2)	
**Education** Below Secondary	10(21.7)	22(47.8)	14(30.5)	0.400
Secondary and above	11(25.0)	25(56.8)	8(18.2)	
**Occupation** Unemployed	15(23.1)	34(52.3)	16(24.6)	0.995
Employed	6(24.0)	13(52.0)	6(24.0)	
**Marital status** Married	14(23.7)	31(52.6)	14(23.7)	0.975
Others	7(22.6)	16(51.6)	8(25.8)	
**Grades of lymphedema** Grade Ⅱ	6(20.0)	18(60.0)	6(20.0)	0.268
Grade Ⅲ	7(23.3)	18(60.0)	5(16.7)	
Grade Ⅳ	8(26.7)	11(36.7)	11(36.6)	
**No. of visits to clinic in the previous year** ≤ 3 times	9(14.8)	34(55.7)	18(29.5)	**0.015**
> 3 times	12(41.4)	13(44.8)	4(13.8)	
Comorbidity* Yes	9(21.4)	21(50.0)	12(28.6)	0.689
No	12(25.0)	26(54.2)	10(20.8)	

*Comorbidities include diabetes, hypertension, thyroid and cardiac diseases.

Values are presented as number (percentage). P values were calculated using the chi-square test.

## Discussion

Though MMDP practices are the major interventions to prevent ADL attacks and disease progression among patients with filarial lymphedema, there is no standard scoring system to rate individual patient’s adherence to the prescribed practices. To address this lacuna, a scoring system was developed, the VCRC MMDP Adherence Assessment Tool and the VAAT score and was validated among patients attending a filarial lymphedema clinic. This tool and the VAAT score have been developed taking into consideration of eight different domains of MMDP practices and the tool is useful for an objective evaluation of the patient’s adherence. Although antibiotic and antifungal creams are included in the MMDP kit, their use is primarily recommended during episodes of secondary infection and is not intended for routine daily application and was not considered in the tool. Apart from the overall adherence, the tool was able to reflect the domain-wise adherence as well. Clinicians can readily identify domains requiring attention and provide targeted guidance to patients accordingly.

The tool was designed to incorporate eight key domains of MMDP practices and enables objective assessment of both overall and domain-specific adherence. In addition to providing a composite adherence score, the tool facilitates identification of specific domains requiring reinforcement, thereby allowing clinicians to deliver targeted counselling during follow-up visits. The findings of this study demonstrate the feasibility and preliminary reliability of the VAAT score in assessing adherence to MMDP practices. While the tool shows promise for use in clinical follow-up, its effectiveness in improving patient outcomes and its impact on long-term implementation were not directly evaluated. Therefore, these findings should be interpreted cautiously. Further studies are required to assess the clinical utility, implementation feasibility, and impact of the tool on morbidity outcomes before its routine adoption in programmatic settings.

In previous studies conducted in different places in India patient’s adherence to MMDP practices was evaluated in a subjective manner using questionnaires; no scoring system has been proposed for an objective evaluation. For instance, in a study done in Villupuram district, Tamil Nadu 100 patients with filarial lymphedema were examined using a structured, pretested questionnaire. Among them 73% were found to practice the leg hygiene measures. However, only 34.2% practiced limb hygiene as instructed [11]. In another study conducted in Odisha, patients from two different places (100 patients from Khurda and 108 patients from Bhubaneswar) were interviewed about the six most widely prescribed foot care procedures. A significant portion of patients were consistently cleaning the afflicted limb. However, other foot care practices like, massaging, elevation and exercising of affected limb and use of bandage were practiced only by a few [12]. In a study by De Backer et al (2015), in Pondicherry, South India conducted on 35 participants, adherence to MMDP practices like limb massaging and usage of crepe bandages were found to be sub-optimal [13].

In the past, adherence rating scales have been developed and used in different scenarios, a common example is evaluation of compliance to exercise program. For instance, an adherence rating scale has been used by Beinart et al. for evaluation of adherence to exercise program. They utilized a five-point Likert scale (0 = Completely agree to 4 = Completely disagree) and the overall score ranges from 0 to 64. They found that a higher overall score indicates a better adherence to the exercises prescribed [14]. Similarly, in our study, a higher score correlated with a better adherence of the patient to MMDP practices.

Several barriers to adherence to MMDP practices were observed during the study. Common patient-related barriers included lack of awareness regarding the long-term benefits of regular self-care, difficulty in maintaining daily routines, physical discomfort during exercises or bandaging and competing occupational and household responsibilities. Socioeconomic constraints, limited access to MMDP supplies and financial limitations also affected consistent practice of recommended measures.

Health system–related barriers included irregular follow-up visits, limited time for individualized counselling and inadequate reinforcement of self-care practices during routine clinic visits. Cultural beliefs, stigma associated with visible limb swelling, and low perceived severity of the disease in early stages further contributed to suboptimal adherence.

Addressing these barriers through structured counselling, community-based follow-up, involvement of caregivers and improved availability of MMDP supplies may enhance long-term adherence and clinical outcomes.

During follow-up visits, the tool facilitated systematic assessment of adherence across multiple MMDP domains and helped to identify specific practices requiring reinforcement or counselling. Additionally, the tool needs to be validated prospectively in different geographic and epidemiological settings, and the factors associated with adherence need to be meticulously evaluated, as well as the clinical outcomes like ADL attacks and progression of lymphedema. The tool was developed to support physicians during follow-up visits, and may require further modification for use by healthcare workers, caregivers or patients.

### Limitations

The study has a number of limitations due to its exploratory-pilot nature. The study relied on self-reported data, which is subject to recall and social desirability bias. Future research should incorporate more objective adherence measures, such as direct observation, periodic photographic documentation of limb care routines, electronic monitoring or caregiver logs to validate self-reported behaviors. Such methods, though resource-intensive, could be used for cross-validation of the VAAT tool and strengthen its utility in both clinical and community settings. This study represents the initial development and pilot validation of the VAAT score. Although the tool demonstrated acceptable internal consistency, comprehensive psychometric evaluation was beyond the scope of the present study. Construct validity, criterion validity, test–retest reliability, sensitivity, specificity, and factor structure were not assessed. Further multicentre studies with larger sample sizes are required to evaluate these psychometric properties and establish the reliability, validity, and generalizability of the VAAT score before its routine clinical or programmatic application. Further studies should include patients with bilateral limb involvement as well, to evaluate the applicability of the VAAT score across a broader spectrum of disease presentations. Additionally, the tool needs to be validated prospectively in different settings, including primary health-care facilities, secondary and tertiary care hospitals, community-based MMDP programs, and geographically diverse endemic regions. The efficacy and robustness of the tool in predicting the progression of the disease can be assessed through a longitudinal study involving a cohort. Objective clinical outcomes, such as changes in disease severity and frequency of ADL attacks, were not assessed in the present study. Inclusion of these parameters in future prospective studies would help establish the relationship between VAAT scores and clinical outcomes and further strengthen the validity of the tool.

## Supporting information

S1 File VAAT scoring questionnaire. (DOCX)

S2 File De-identified datasheet. (XLSX)

## Abbreviations

LF: Lymphatic Filariasis
GPELF: Global Programme to Eliminate Lymphatic Filariasis
MDA: Mass Drug Administration
MMDP: Morbidity Management and Disability Prevention
NTD: Neglected Tropical Diseases
ADL: Adenolymphangitis

## References

[1] World Health Organization. Lymphatic filariasis. World Health Organization. 2022. https://www.who.int/news-room/fact-sheets/detail/lymphatic-filariasis Accessed 2022 July 28.

[2] Pani S, Das PK, Krishnamoorthy K. Prospects of elimination of lymphatic filariasis in India. ICMR Bulletin. 2022;32(5 & 6):41–54.

[3] National Center for Vector Borne Diseases Control (NCVBDC). Elephantiasis magnitude of disease. 2022. https://ncvbdc.mohfw.gov.in/index4.php?lang=1&level=0&linkid=455&lid=3732 Accessed 2023 May 16.

[4] Akhter S. 40% of the global burden of lymphatic filariasis is shared by India: Prof. Dr. Suma Krishnasastry. ET Health World. 2018. https://health.economictimes.indiatimes.com/news/industry/40-of-the-global-burden-of-lymphatic-filariasis-is-shared-by-india-prof-dr-suma-krishnasastry/64892424

[5] World Health Organization. Global programme to eliminate lymphatic filariasis: progress report, 2022. Wkly Epidemiol Rec. 2023;98(41):489–502.

[6] Bizhani N, Hashemi Hafshejani S, Mohammadi N, Rezaei M, Rokni MB. Lymphatic filariasis in Asia: a systematic review and meta-analysis. Parasitol Res. 2021;120(2):411–22. doi: 10.1007/s00436-020-06991-y 33415391 PMC7790732

[7] Addiss DG, Brady MA. Morbidity management in the Global Programme to Eliminate Lymphatic Filariasis: a review of the scientific literature. Filaria J. 2007;6:2. doi: 10.1186/1475-2883-6-2 17302976 PMC1828725

[8] Dreyer G, Addiss D, Dreyer P, Noroes J. Basic lymphoedema management: treatment and prevention of problems associated with lymphatic filariasis. 2nd ed. Recife: Amaury Coutinho Foundation. 2002.

[9] World Health Organization. Morbidity management and disability prevention (MMDP). https://www.who.int/activities/building-capacity-of-national-programmes-to-implement-who-recommended-strategies/morbidity-management-and-disability-prevention. Accessed 2022 October 18.

[10] Basavaraj K, Bharatesh SK, Murali D, Ramachandra K, Sowmya M. A Study on Morbidity Management among Lymphatic Filariasis Patients in Udupi district, Karnataka, India. IJMEDPH. 2017;7(2):92–6. doi: 10.5530/ijmedph.2017.2.18

[11] Kumari AK, J Y, Das LK. Issues in delivering morbidity management for lymphatic filariasis elimination: a study in Pondicherry, South India. ScientificWorldJournal. 2012;2012:372618. doi: 10.1100/2012/372618 22654597 PMC3361224

[12] Bontha VB, Abhay NN, Anna SK. A survey on foot care practices among filarial lymphoedema patients in Orissa, India. Trop Biomed. 2007;24(2):7–14. 18209702

[13] DeBacker H, Vijesh Sreedhar K. Health-seeking behavior and morbidity management practices among chronic lymphatic filariasis patients in South India. Puducherry: ICMR-Vector Control Research Centre.

[14] Newman-Beinart NA, Norton S, Dowling D, Gavriloff D, Vari C, Weinman JA, et al. The development and initial psychometric evaluation of a measure assessing adherence to prescribed exercise: the Exercise Adherence Rating Scale (EARS). Physiotherapy. 2017;103(2):180–5. doi: 10.1016/j.physio.2016.11.001 27913064

