# Supplementary material for: Developing a scoring tool for assessing adherence to morbidity management and disability prevention (MMDP) practices in patients with filarial lymphedema: A pilot study
Source: PLoS One. 2026 Sep 30;21(9):e0358191. doi: 10.1371/journal.pone.0358191 (PMC13626310; doi:10.1371/journal.pone.0358191)
Supplement: Supplementary file 1 [file pone.0358191.s001.docx]

**Supporting Information:**

**S1 Table: VAAT Scoring Questionnaire**

| **Socio-demographic details**:  1. Name: | | | | | | |
| --- | --- | --- | --- | --- | --- | --- |
| 2. Patient Registration ID and Date: | | | | | | |
| 3. Age: | | | | | | |
| 4. Address: | | | | | | |
| 5. Phone No: | | | | | | |
| 6. Gender: 1. Male 2. Female 3. Others | | | | | | |
| 7. Education: | | | | | | |
| 8. Occupation: | | | | | | |
| 9. Marital status:  **Clinical details:** | | | | | | |
| 10. Lymphedema grade: | | | | | | |
| 11. Affected body part: | | | | | | |
| 12. Duration of illness: | | | | | | |
| 13. How many times have you had ADL attacks in the last year? 14. History of ADL attack: | | | | | | |
| 15. When did you have your last ADL attack: 16. Co-morbidities condition: | | | | | | |
| **Limb Hygiene** | | | | | | |
| 1 | Do you wash your legs as a practice of MMDP? | **Yes =1** | | **No =0** | | |
| 2 | How often do you wash your legs in a day? | **Once = 1** | | **Twice = 2** | | |
| 3 | Do you wash both legs even if one leg is affected? | **Yes =1** | | **No =0** | | |
| 4 | Do you use soap to wash your legs? | **Yes =1** | | **No =0** | | |
| 5* | Are you using coconut fibres/brushes for cleansing? | **Yes =0** | | **No =1** | | |
| 6 | Do you wash your legs above knee to foot direction? | **Yes =1** | | **No =0** | | |
| 7 | Do you clean the interdigital spaces and skin folds? | **Yes =1** | | **No =0** | | |
| 8 | Do you examine your skin for intertrigo (Whitish patches in-between the toes)? | **Yes =1** | | **No =0** | | |
| 9 | Do you use towel/cotton cloth for drying your legs? | **Yes =1** | | **No =0** | | |
| 10 | Are you using the towel gently? | **Yes =1** | | **No =0** | | |
| 11 | Do you use cotton cloth or gauze to dry interdigital spaces or skin folds? | **Yes =1** | | **No =0** | | |
| **Limb Elevation** | | | | | | |
| 12 | Do you elevate the legs as a part of MMDP? | **Yes =1** | | **No =0** | | |
| 13 | Do you raise the legs up to the hip whenever sitting for long time? | **Yes =1** | | **No =0** | | |
| **Limb exercise** | | | | | | |
| 14 | Do you practice limb exercise? | **Yes =1** | | **No =0** | | |
| 15 | Do you practice, “stand on your toes limb exercise”? | **Yes =1** | | **No =0** | | |
| 16 | Do you practice, “toe point limb exercise”? | **Yes =1** | | **No =0** | | |
| 17 | Do you practice, “circle foot exercise”? | **Yes =1** | | | | **No =0** |
| 18 | Do you practice, “cycling movement limb exercise”? | **Yes =1** | | | | **No =0** |
| 19* | Do you practice exercise while you suffer from fever, chills, limb pain or redness also? | **Yes =0** | | | | **No =1** |
| 20 | How often do you practice limb exercises in a day? | **Once = 1** | | | | **Twice = 2** |
| **Wearing appropriate footwear** | | | | | | |
| 21 | Are you using footwear? | **Yes =1** | | | | **No =0** |
| 22° | Are you using comfortable footwear with proper size? | **Yes =1** | | | | **No =0** |
| 23* | Do you wear footwear when blisters or wound is present? | **Yes =0** | | | | **No =1** |
| **Wearing crepe bandage or stockings** | | | | | | |
| 24 | Do you wear crepe bandage or stockings? | **Yes =1** | | | | **No =0** |
| 25 | Do you wear that throughout the day? | **Yes =1** | | | | **No =0** |
| 26° | Do you wear crepe bandage “foot to the knee direction”? | **Yes =1** | | | | **No =0** |
| 27* | Do you use crepe bandage while you suffer from fever, chills, limb pain or redness also? | **Yes =0** | | | | **No =1** |
| **Massaging** | | | | | | |
| 28 | Do you practice massaging as a part of MMDP? | | **Yes =1** | | **No =0** | |
| 29 | How often do you practice massaging in a day? | | **Once = 1** | | **Twice = 2** | |
| 30 | Are you massaging in “foot to knee direction”? | | **Yes =1** | | **No =0** | |
| 31 | Do you wear crepe bandage or stockings soon after massaging? | | **Yes =1** | | **No =0** | |
| 32 | Do you massage while you suffer from fever, chills, limb pain or redness also? | | **Yes =0** | | **No =1** | |
| **Nail care** | | | | | | |
| 33 | Do you cut your nails once a week? | | **Yes =1** | | **No =0** | |
| 34 | While cutting nails do you take precautions, not to injure the toe or the skin? | | **Yes =1** | | **No =0** | |
| **Application of liquid paraffin** | | | | | | |
| 35 | Do you apply liquid paraffin after washing and drying your skin? | | **Yes =1** | | **No =0** | |
| 36 | How often do you apply liquid paraffin in a day? | | **Once = 1** | | **Twice = 2** | |

*Negative questions

°Examined by the investigator
